# Clinical Conditions Associated With a High Antinuclear Antibody Titer in Individuals Without Autoimmune Disease

**DOI:** 10.1002/acr.25682

**Published:** 2026-01-20

**Authors:** Matthew Chung, John P. Shelley, Gul Karakoc, John Still, Xiaodi Ruan, Jonathan Mosley, C. Michael Stein, Vivian K. Kawai

**Affiliations:** ^1^ Vanderbilt University Nashville Tennessee; ^2^ Vanderbilt University Medical Center Nashville Tennessee; ^3^ University of Texas Southwestern Medical Center Dallas; ^4^ University of Alabama at Birmingham

## Abstract

**Objective:**

Antinuclear antibodies (ANAs) are present at high titers in 2% of the general population, but their clinical significance in individuals without an autoimmune (AI) disease is not known. We tested the hypothesis that the presence of a high ANA titer in non‐AI conditions is associated with disease.

**Methods:**

We conducted a retrospective case‐control study in the Vanderbilt University Medical Center's de‐identified electronic medical record system. Individuals without AI disease who had an ANA test were classified into three groups: high titer (HT; ANA ≥ 1:640), low titer (LT; ANA ≤ 1:80), and negative (NG) ANA results. The prevalence of diagnoses recorded within 90 days of the ANA test were compared among groups in a phenome‐wide association study adjusting for age at ANA testing, sex, median body mass index (BMI), and reported race. A *P* value <5 × 10^−5^ was considered significant.

**Results:**

A total of 28,781 individuals qualified for the study: 3.1% in the HT, 12.3% in the LT, and 84.6% in the NG groups. BMI was similar among groups (*P* value = 0.345), but individuals in the HT group were older (*P* = 3.9 × 10^−73^). A high ANA titer increased risk of 46 and 67 clinical diagnoses when comparing the HT group with the LT and the NG groups, respectively. The most significant associations in both comparisons included liver disorders and complications and risk factors for liver disease.

**Conclusion:**

A high ANA titer in the absence of an AI disease was associated with increased risk of liver disorders and related risk factors and complications.

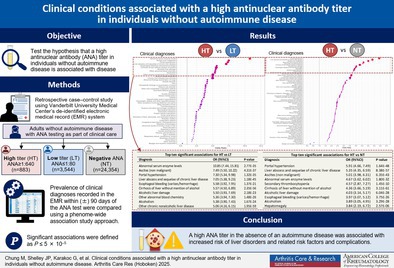

## INTRODUCTION

Antinuclear antibodies (ANAs) represent a diverse group of autoantibodies that bind to specific molecular components within the nucleus of a cell. Their presence in blood serves as an important diagnostic tool in clinical care^1^ because it provides important diagnostic information through the resulting patterns and titers.^2^

**SIGNIFICANCE & INNOVATIONS**

- This is the first large study that systematically evaluates the impact of a high antinuclear antibody (ANA) titer (≥1:640) in individuals without an autoimmune disease using a high‐throughput approach.
- A high ANA titer is strongly associated with liver‐related disorders including nonalcoholic and alcoholic liver disease in individuals without autoimmune disease.
- Nonautoimmune liver disease should be included in the differential diagnosis in patients who have an ANA titer ≥1:640.

ANAs are biomarkers commonly used in the diagnosis of several autoimmune (AI) diseases,^1^ with a positive ANA test being almost mandatory for the diagnosis of systemic lupus erythematosus (SLE)^3^ because more than 95% of patients with SLE test positive and the classification criteria for SLE require an ANA titer of at least 1:80.^3^ A positive ANA test is also associated with an increased risk of AI conditions other than SLE, such as Sjögren disease, systemic sclerosis, mixed connective tissue disease, AI liver disease, and idiopathic inflammatory myopathies among others.^1^

Although ANAs are well known for their association with various AI diseases, their occurrence in the general population suggests broader biologic implications.^4^ A small study reported that healthy individuals with ANA^+^ (≥1:120) showed suppression of T cell signatures and lower cytokine levels compared to their counterpart ANA^−^ individuals^4^, ^5^; but it is unclear whether this altered immunologic landscape might contribute to the development or prevention of various clinical conditions.

Some clinical studies have indicated that individuals with a positive ANA test, particularly at higher titers,^6^ have an increased risk of cardiovascular events,^6^ cancer,^7^ infections,^8^ and all‐cause mortality^6^, ^9^; but these findings have not been consistent.^10^, ^11^ Recently, we showed that a positive ANA test at a titer ≥1:80 in individuals without an AI disease was significantly associated with increased risk for Raynaud phenomenon and idiopathic fibrosing alveolitis^12^; however, the clinical implications of a high ANA titer in people without AI disease is unclear.

We hypothesized that individuals without AI disease who have a high ANA titer are more likely to have immune dysregulation that would manifest as differential disease risk. To test this hypothesis, we used a phenome‐wide association study (PheWAS) approach to identify clinical diagnoses that are enriched in patients without AI disease who have a high ANA titer compared to individuals with a low titer and negative ANA results.

## PATIENTS AND METHODS

### Study population

The study was approved by the Vanderbilt University Medical Center (VUMC) Institutional Review Board. We selected individuals from the de‐identified copy of the electronic medical record (EMR). Participants were 18 years or older and had at least one clinician‐ordered ANA test. Because titers were reported in ≥95% of participants with a positive ANA test since 2000, only ANA results recorded between January 2000 and October 2022 were included. Only ANA tests performed using indirect immunofluorescence of human epithelial type 2 (HEp‐2) cells were selected. The hospital clinical laboratory used protocols recommended by the American College of Rheumatology Task Force^13^ using anti‐human IgG conjugated in HEp‐2 cells from Immuno Concepts and Inova Diagnostics laboratory. Testing was performed following manufacturer's recommendations, and while manual interpretation was performed in both assays, the Inova Diagnostics assay also provided automated interpretation.^14^

For individuals with more than one eligible ANA result, we selected the test with the highest titer. We excluded any individual with a diagnosis of an AI disorder commonly considered to be associated with a positive ANA test (Supplementary Table S1).^1^ To define the clinical impact of a high ANA titer, eligible participants were divided into three mutually exclusive groups: (a) high titer (HT) included individuals with an ANA titer ≥1:640,^15^ (b) low titer (LT) included individuals with ANA titer ≤1:80, and (c) negative (NG) included individuals who only had negative ANA results in their EMR. Individuals with a titer between 1:80 and 1:640 were excluded from the analyses.

### Covariates

Demographic and clinical data including age at test, sex, reported race, and body mass index (BMI; kg/m^2^) were extracted from the EMR and used as covariates. The median BMI was calculated using plausible BMIs recorded in the EMR, which we defined as being between 15 and 60 as previously used.^16^ Only participants with data available for all covariates were included in the analyses.

### Statistical analysis

To define the clinical impact of a high ANA titer, the prevalence of different clinical diagnoses was compared between HT and LT groups and between the HT and NG groups using a PheWAS approach,^17^ which is a method for high‐throughput analyses using EMR data. To define which clinical diagnoses were more likely associated with a high ANA titer, only diagnostic codes first recorded within 90 days of the selected ANA results were included. International Classification of Diseases (ICD) codes (versions 9 and 10) were extracted from the EMR and transformed into Phecodes, which aggregate one or more related ICD codes into distinct diseases or traits.^18^ The ICD‐to‐Phecode map (version 1.2) was used to create Phecodes. For each clinical diagnosis, cases were defined as individuals with two or more instances of the specific Phecode in the EMR. Controls were defined as individuals without the Phecode or any closely related Phecode. Analyses were adjusted for age at ANA testing, sex, median BMI, and race. Only Phecodes with 100 cases or more were analyzed to assure power. PheWAS was performed using the PheWAS R package,^17^ and associations with a *P* value <5 × 10^−5^ were considered significant. Continuous and categorical variables are shown as median (interquartile range [IQR]) and frequency (percentage), respectively, and compared using a Kruskal–Wallis rank test and a chi‐squared test.

### Case validation

Because the quality of phenotype definition varies when ICD codes are aggregated, the medical record of a random sample of 50 individuals for the 10 most significant associations was manually reviewed to confirm the diagnosis and estimate the positive predictive value (PPV) for the selected Phecodes. If related Phecodes were among top associations, the Phecode with the highest number of cases was selected for chart review.

### Data availability

The data sets generated and/or analyzed during the current study are available from the corresponding author on reasonable request. Phecode maps and R code for the PheWAS are openly available at https://phewascatalog.org/phewas.

### Ethics approval and consent to participate

The study was approved by the VUMC Institutional Review Board; the study met Exemption 4 criteria and is not considered human participant research under the 2018 Revised Common Rule requirement of the Office for Human Research Protections. Thus, consent form was not required. Data used for the study come from the de‐identified EMR system at VUMC. No experiments in humans were performed as part of the study, de‐identified data that were already collected were used for in silico analysis, and all methods were performed in accordance with relevant guidelines and regulations. The authors did not have direct contact or knowledge of the individuals’ identities.

## RESULTS

**Figure 1:**
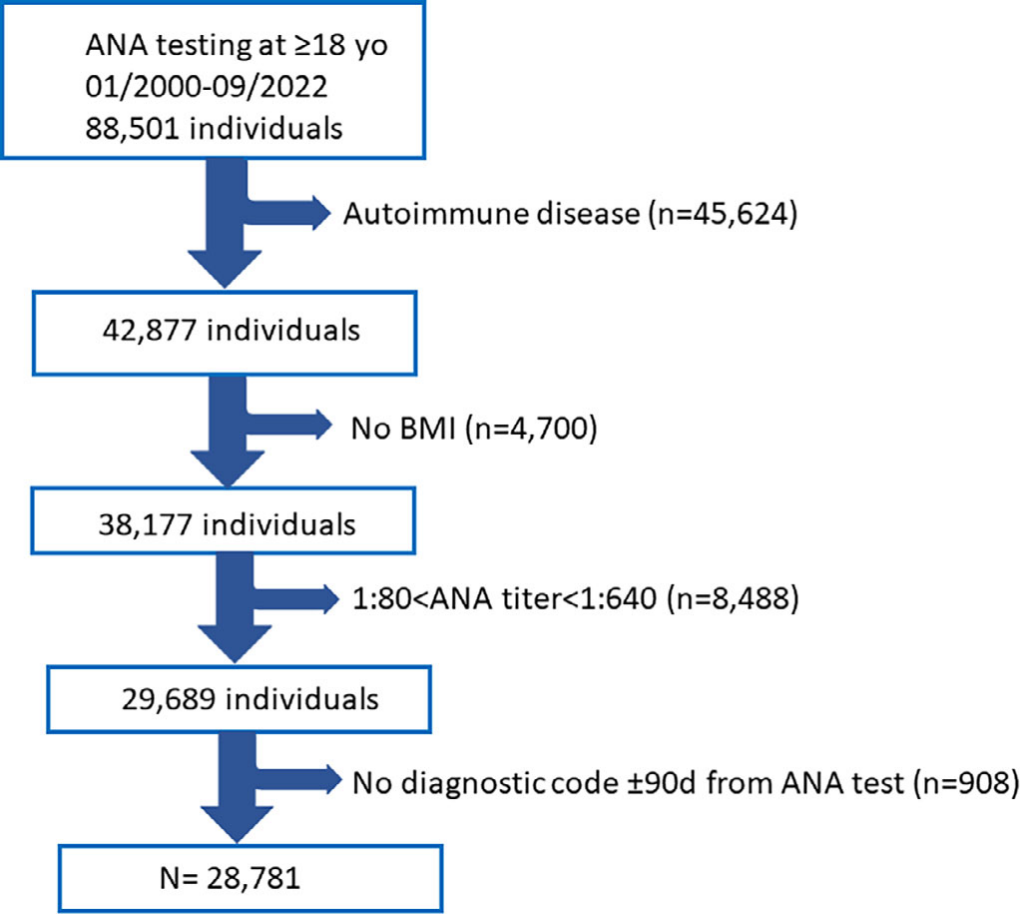
Flowchart of the study showing selected individuals with an ANA test result at 18 years or older in the Vanderbilt University Medical Center EMR system. ANA, antinuclear antibody; BMI, body mass index; EMR, electronic medical record; yo, years old. Color figure can be viewed in the online issue, which is available at http://onlinelibrary.wiley.com/doi/10.1002/acr.25682/abstract.

Between January 2000 and September 2022, there were 88,501 participants who were 18 years or older and had at least one clinician‐ordered ANA test available. We excluded individuals with a diagnosis of an ANA‐related AI disease in their EMR (n = 45,624), those without BMI information (n = 4,700), and those with an ANA titer higher than 1:80 but lower than 1:640 (n = 8,488). There were 29,689 individuals with an eligible ANA result, and 28,781 of them had at least one diagnosis code first recorded within ±90 days of the eligible ANA result (Figure 1 shows the flowchart of the study design).

Table 1 shows the demographic and clinical characteristics of the 28,781 eligible individuals: 3.1% (n = 883) were in the HT, 12.3% (n = 3,544) in the LT, and 84.6% (n = 24,354) in the NG groups, respectively. Overall, the study population was largely of European ancestry, and there were more women. Individuals in the HT groups were older (median age 55 [IQR 42, 66] years) than those in the LT (median 48 [IQR 37, 60] years) and NG (median 45 [IQR 33, 56] years) groups, and the proportion of White individuals was smaller. BMI did not differ significantly among the three groups (median 28.7 [IQR 24.5, 33.3] kg/m^2^, median 28.1 [IQR 24.2, 33.3] kg/m^2^, and median 28.2 [IQR 24.1, 33.4] kg/m^2^ for the HT, LT, and NG groups, respectively). An ANA pattern was available in 23.4% and 95.8% of the individuals in the LT and HT groups, respectively, and the distribution of patterns was very similar in both groups (Table 1).

**Table 1:** Characteristics of study population categorized by their ANA result*

Characteristics	Negative	Low titer	High titer
	(n = 24,354)	(n = 3,544)	(n = 883)
Age at testing, y^a^	45.0 (33.0, 56.0)	48.0 (37.0, 60.0)	55.0 (42.0, 66.0)
Female, n (%)	15,912 (65.3)	2,577 (72.7)	613 (69.4)
Body mass index,^a^ kg/m^2^	28.2 (24.1, 33.4)	28.1 (24.2, 33.3)	28.7 (24.5, 33.3)
Reported race, n (%)			
White	17,779 (73.0)	2,643 (74.6)	596 (67.5)
Black	2,633 (10.8)	326 (9.2)	85 (9.6)
Asian	442 (1.8)	52 (1.4)	11 (1.3)
Hispanic	617 (2.5)	77 (2.2)	15 (1.7)
Other/unknown	2,883 (11.8)	446 (12.6)	176 (19.9)
ANA pattern,^b^ n (%)		(n = 830)	(n = 846)
Homogeneous	NA	476 (57.3)	478 (56.5)
Speckled	NA	309 (37.2)	329 (38.9)
Nucleolar	NA	42 (5.1)	28 (3.3)
Others	NA	3 (0.4)	11 (1.3)

* ANA, antinuclear antibody; NA, not applicable.

^a^ Age at testing and body mass index are shown as median (interquartile range).

^b^ ANA patterns were present in 23% and 95.8% of patients in the low (ANA titer ≤ 1:80) and high titer (ANA titer ≥ 1:640) groups, respectively.

### Clinical associations comparing HT and LT groups

**Figure 2:**
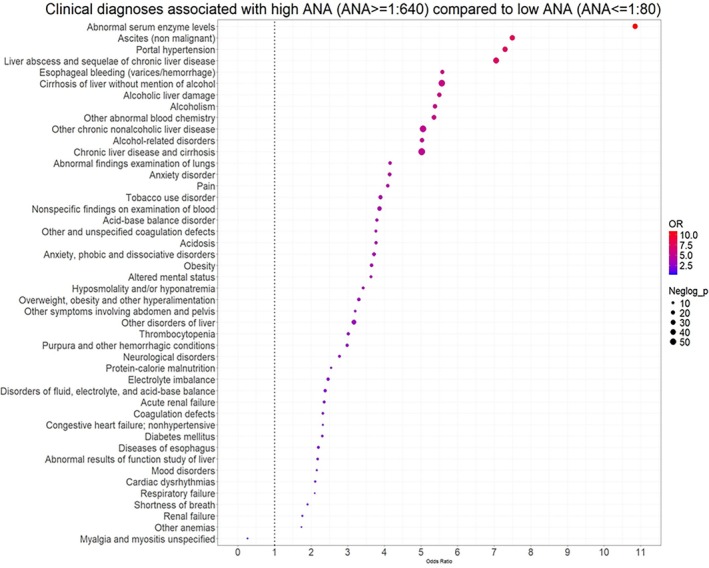
Significant clinical associations when individuals with high titers (ANA ≥ 1:640) are compared with individuals with low ANA titers (ANA ≤ 1:80). The magnitude (OR) and strength (*P* value) of the association are represented by the circle size and color, respectively. The dotted vertical line represents no association, and circles on the left and right from the dotted line represent decreased and increased risk, respectively. ANA, antinuclear antibody; OR, odds ratio.

Individuals in the HT group had increased risk of 46 clinical Phecode diagnoses (Figure 2). Top associations were chronic liver diseases (Phecode 571), nonalcoholic fatty liver disease or steatohepatitis (NAFLD/NASH; Phecode 571.5), and alcohol‐related liver disease (Phecode 317). Other significant associations were related to other liver disorders and their complications, disorders known to increase liver disease (alcoholism, metabolic and biliary disorders), and respiratory, gastrointestinal, and mood disorders (Supplementary Table S2). A Phecode (338) for pain was more common in the HT group compared to the LT group (odds ratio [OR] = 4.1, *P* value = 1.9 × 10^−13^), but myalgia (Phecode 770) was less common (OR = 0.27, *P* value = 1.3 × 10^−5^).

### Clinical associations comparing HT and NG groups

**Figure 3:**
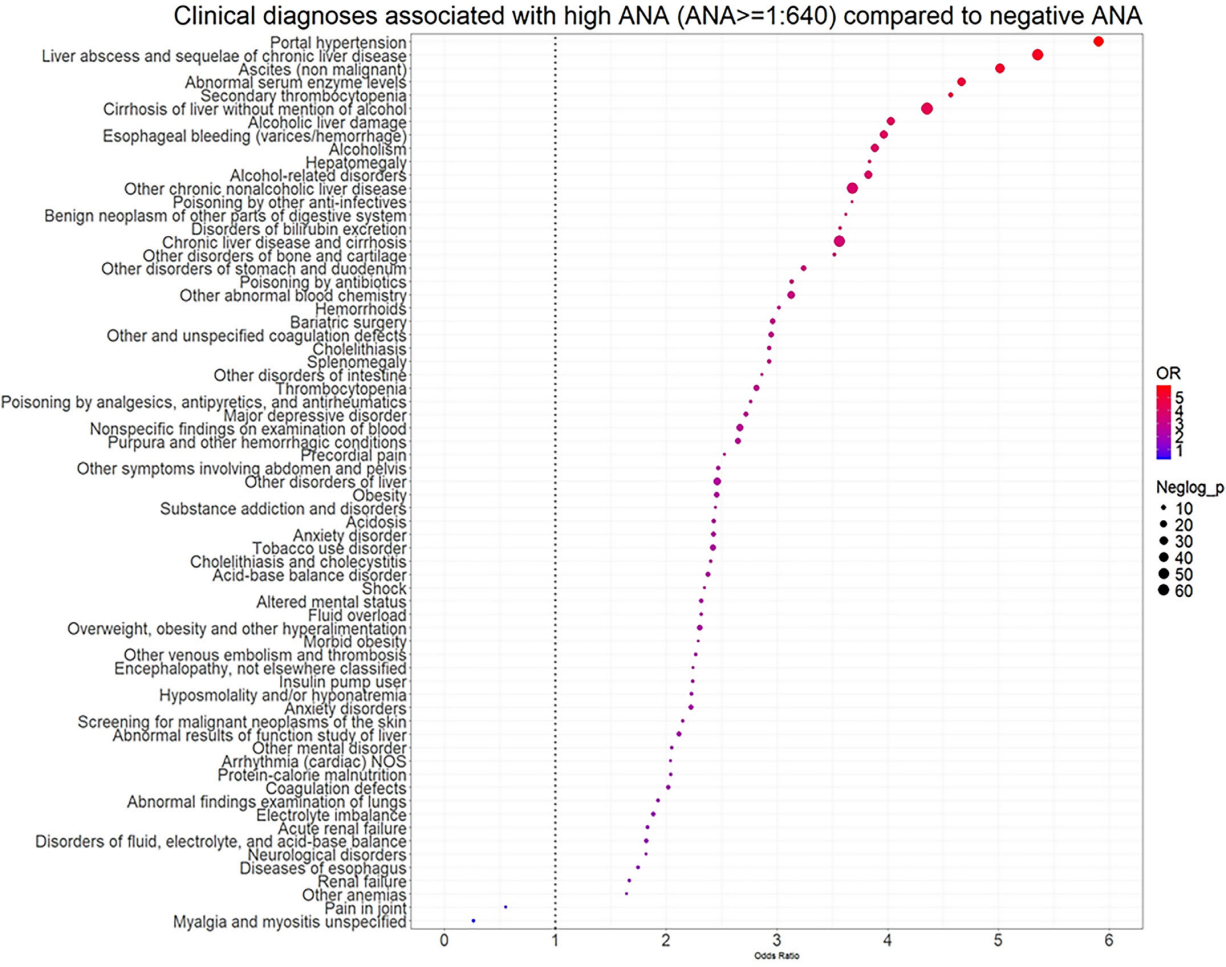
Significant clinical associations when individuals with high ANA titers (ANA ≥ 1:640) are compared with individuals with a negative ANA test. The magnitude (OR) and strength (*P* value) of the association are represented by the circle size and color, respectively. The dotted vertical line represents no association, with circles on the left and right from the dotted line representing decreased and increased risk, respectively. ANA, antinuclear antibody; NOS, not otherwise specified; OR, odds ratio.

When compared to individuals with a negative ANA test, 67 clinical diagnoses were more prevalent in the HT group (Figure 3). Fifty‐nine of these corresponded to diagnoses or closely related diagnoses—morbid obesity, bariatric surgery, substance disorders for alcoholism, and insulin pump usage—that were also observed in the comparison between the HT and LT groups. Top associations included the same liver disorders observed when comparing HT with LT groups. New associations included increased risk for gastrointestinal disorders, biliary tract disorders, poisoning by different agents (such as antibiotics, other anti‐infectives, and analgesics/antipyretics/antirheumatics), and screening for skin cancers (Supplementary Table S3).

A sensitivity analysis excluding BMI as a covariate showed similar results when comparing the HT with the LT group (Supplementary Table S4) and the NG group (Supplementary Table S5). Among individuals in the HT group, ANA patterns were not different between those with and without liver disease (Supplementary Table S6).

### Case validation

Because the most significant clinical diagnoses associated with high ANA titers were liver‐ and alcohol‐related disorders, we selected 50 random cases with a high ANA titer and Phecode 573 (“Other disorders of the liver”) to perform chart review. We confirmed the presence of non‐AI liver disease in 44 individuals (PPV of 88%) with 3 individuals having possible primary biliary cholangitis, primary biliary cirrhosis, or AI hepatitis. Fatty liver‐related diseases were the most common source for liver disease followed by alcohol consumption and hepatitis C infection. Another top association was related to alcohol disorder (alcoholism—Phecode 317.1 and alcohol liver damage—Phecode 317.11), with 46 of the 50 random charts reviewed for these categories indicating diagnoses attributed to alcohol consumption (PPV = 92%).

## DISCUSSION

A striking finding was that a high ANA titer is strongly associated with liver‐related disorders such as NAFLD and NASH as well as alcoholic liver disorders. High titers of ANAs are uncommon in people without AI disease^10^, ^19^; however, few studies have examined the clinical implications of these high ANA titers.^15^, ^20^ Although ANA titers (>1 SD above the mean in healthy controls) are associated with upregulation of type I tumor necrosis factor signatures,^20^ little is known about their associations with disease.

In a laboratory‐based study, 5% of individuals tested had ANA titers ≥1:640, and 44% of them did not have a diagnosis of an AI disorder.^15^ Among these individuals with high ANA titer without AI disease, 43% and 24% of them returned for a 5‐year and 10‐year follow‐up, respectively.^21^, ^22^ Although ANA results remained positive in 91% of those who returned at a 5‐year follow‐up, only 3 of 62 participants had a new diagnosis of an AI disease.^21^ As for those who returned for a 10‐year follow‐up, 75% continued to have positive ANA results, and 2 of 34 participants were diagnosed with an AI disease at 10‐year follow‐up.^22^ These findings suggest that the majority of high titer positive ANA results are not explained by the future development of an AI disease.

Using the results of ANA testing performed as part of routine clinical care, we defined the associations between a high titer positive ANA test and disease in a large population that did not have AI disease. The most striking finding was the increased prevalence of liver disease and diagnoses related to liver disease, particularly NAFLD and NASH. An association between a positive ANA test and AI disease is well‐recognized,^1^ and we had therefore excluded people with liver diseases such as AI hepatitis and primary biliary cholangitis from the study cohort; review of a sample of medical records showed that undiagnosed AI liver disease did not account for our findings.

Also, a high ANA titer was associated with increased risk of alcoholic liver disease (ALD) and alcohol consumption, suggesting that liver injury of differing etiology can predispose to a high titer ANA. The finding that high ANA titers were associated with increased risk of alcohol‐ and tobacco‐related disorders is in agreement with previous clinical and animal studies that suggested increased formation of autoantibodies,^23^ including ANAs,^24^ under conditions of increased oxidative stress,^25^ such as tobacco use^26^ and chronic alcohol use.^27^

Although non‐AI liver disease is not currently recognized as a frequent cause of a high titer ANA, a positive ANA test occurs in approximately a third of individuals with NAFLD^28^ in the absence of AI hepatitis.^29^ The relationship between the presence of a positive ANA test and histologic features in the liver of patients with NAFLD is uncertain.^30^, ^31^, ^32^ A small clinical study suggested that a positive ANA test, even at high titer (≥1:640), was not associated with disease activity and severity of histologic changes,^30^ whereas other studies reported an association between positive ANA results and higher levels of hepatic fibrosis and inflammation.^31^, ^32^ We found that, in addition to associations with liver disease, a high titer ANA was associated with increased risk of several diagnoses characteristic of more severe liver disease, including cirrhosis, liver failure, portal hypertension, esophageal bleeding, coagulopathy, ascites, and protein–calorie malnutrition among others. Whether these associations reflect accelerated progression of NAFLD, as suggested by previous studies,^32^, ^33^ cannot be determined in our study.

In addition to liver disease, a high ANA titer was significantly associated with increased risk of several metabolic disorders (overweight, obesity, and diabetes mellitus) or their surrogates (bariatric surgery, insulin pump user). Although the median BMI among the three ANA groups was similar, the percentage of individuals in the HT group that fell in the overweight, obese, and morbidly obese categories was almost twice than that of those assigned to the same categories in the LT and NG groups. A relationship between high ANA titers and metabolic disorders has not been established previously. Cross‐sectional epidemiologic studies suggest an inverse association between BMI and a positive ANA test,^19^, ^34^ which is counterintuitive because obesity is a state of chronic systemic inflammation that could lead to immune dysregulation and obesity is a risk factor for several AI disorders.^35^, ^36^ Although some evidence suggests a positive association between positive ANA results and BMI only at high levels of inflammation,^35^ we cannot exclude the possibility that the association with obesity is driven by NAFLD or NASH.

We found that a high ANA titer was inversely associated with myalgia and pain in joints. This is not surprising considering that individuals with musculoskeletal pain are more likely to be screened for AI disorders with an ANA test, and those with an AI disease are more likely to have a high ANA titer.^36^, ^37^, ^38^

Our study has some limitations: first, some liver disorders associated with a high ANA titer may have been incorrectly assigned to NAFLD or NASH or ALD; however, review of a random selection of medical records showed that these were the most common conditions mentioned in the physician clinical notes. Second, it is possible that some individuals with a high ANA titer will develop an AI disease in the future; to decrease this possibility, we excluded any individuals who had a diagnosis of an AI disease in their EMR at any time, not only before the ANA test. Third, although we tried to capture only incident diagnoses, we cannot exclude the possibility that new patients with chronic conditions diagnosed elsewhere had an ANA test done at the first medical encounter in VUMC, and thus, some chronic conditions were captured. Fourth, because we studied a large cohort of patients from a tertiary care hospital, our findings may not be generalizable to other settings. Fifth, although the International Consensus on Antinuclear Antibody Patterns (ICAP) has released several recommendations for ANA testing,^39^ most of the ANA results used in the analyses were performed before any of these recommendations were released. Sixth, we did not study antibodies associated with DFS70, which is present as a dense fine speckled pattern. These antibodies are difficult to identify using HEp‐2 indirect fluorescent antibody^40^ and require solid phase assays to be detected accurately. Whether the presence of these antibodies may explain some of the observed associations requires further investigation. Nevertheless, our study includes clinical and laboratory results from real‐world patient care encounters from a large university teaching hospital, which is a strength of the study. Other strengths included the use of the EMR system to define a large cohort of individuals tested and limiting the associated diagnoses to those occurring within ±90 days of the ANA, which reduces spurious associations due to prevalent disease. We also adjusted our analyses by sex, age, race, and BMI because these are factors that influence ANA results.

Our study is the first to address the clinical relevance of a high ANA titer in individuals without an AI disease using real‐world data and high‐throughput analyses. Although previous small clinical studies suggested an association between presence of ANA and non‐AI liver disease, our results show that a high ANA titer is associated not only with liver disease but also with complications associated with advanced liver disease. Thus, this suggests that non‐AI liver disease should be considered in differential diagnosis of a high ANA titer. In summary, we found that a high ANA titer (≥1:640) in individuals without an AI disease is more common in patients with liver disease and related sequelae.

## AUTHOR CONTRIBUTIONS

All authors contributed to at least one of the following manuscript preparation roles: conceptualization AND/OR methodology, software, investigation, formal analysis, data curation, visualization, and validation AND drafting or reviewing/editing the final draft. As corresponding author, Dr Kawai confirms that all authors have provided the final approval of the version to be published and takes responsibility for the affirmations regarding article submission (eg, not under consideration by another journal), the integrity of the data presented, and the statements regarding compliance with institutional review board/Declaration of Helsinki requirements.

## Supporting information


**Disclosure form**.

**Table S1:** Phecodes and ICD codes used to exclude ANA‐related autoimmune diseases from the VUMC EMR system.
**Table S2:** Clinical associations for high ANA titer (>=1:640) compared to low ANA titer (<=1:80) in VUMC, adjusted by age at ANA test, sex, race, and BMI.
**Table S3:** Clinical associations for high ANA titer (>=1:640) compared to negative ANA in VUMC, adjusted by age at ANA test, sex, race, and BMI.
**Table S4:** Clinical associations for high ANA titer (>=1:640) compared to low ANA titer (<=1:80) in VUMC, adjusted by age at ANA test, sex, and race.
**Table S5:** Clinical associations for high ANA titer (>=1:640) compared to negative ANA in VUMC, adjusted by age at ANA test, sex, and race.
**Table S6:** ANA pattens for individuals with high ANA titer (≥1:640) with and without liver disease.

AC&R Journal Club
