# Supplementary material for: Clinical Conditions Associated With a High Antinuclear Antibody Titer in Individuals Without Autoimmune Disease
Source: Arthritis Care Res (Hoboken). 2026 Jan 20;78(5):662–9. doi: 10.1002/acr.25682 (PMC13116006; doi:10.1002/acr.25682)
Supplement: Supplementary file 3 — AC&R Journal Club [file ACR-78-662-s002.pptx]

## Slide 1
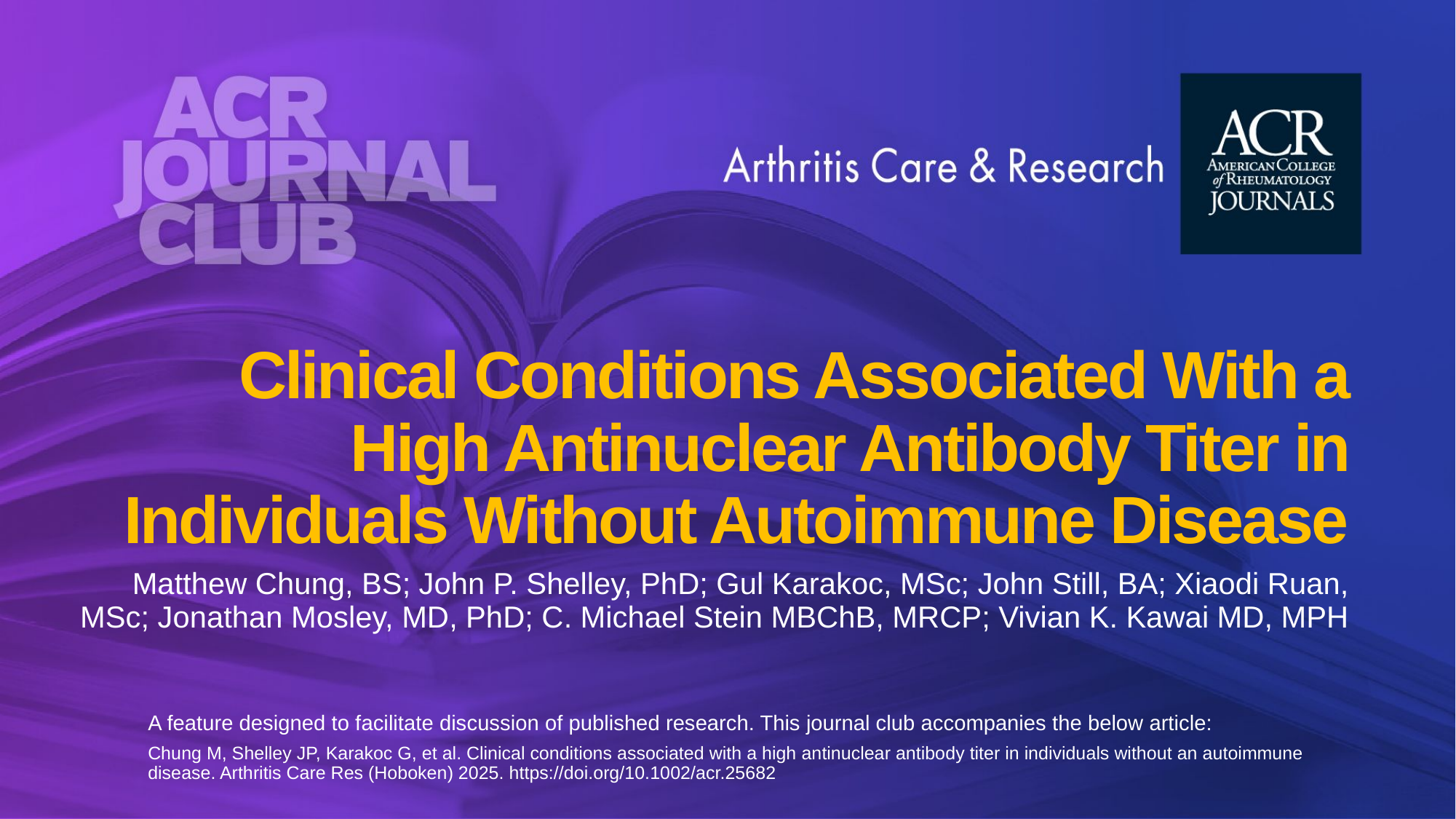

# Clinical Conditions Associated With a High Antinuclear Antibody Titer in Individuals Without Autoimmune Disease
Matthew Chung, BS; John P. Shelley, PhD; Gul Karakoc, MSc; John Still, BA; Xiaodi Ruan, MSc; Jonathan Mosley, MD, PhD; C. Michael Stein MBChB, MRCP; Vivian K. Kawai MD, MPH
A feature designed to facilitate discussion of published research. This journal club accompanies the below article:
Chung M, Shelley JP, Karakoc G, et al. Clinical conditions associated with a high antinuclear antibody titer in individuals without an autoimmune disease. Arthritis Care Res (Hoboken) 2025. https://doi.org/10.1002/acr.25682

## Slide 2
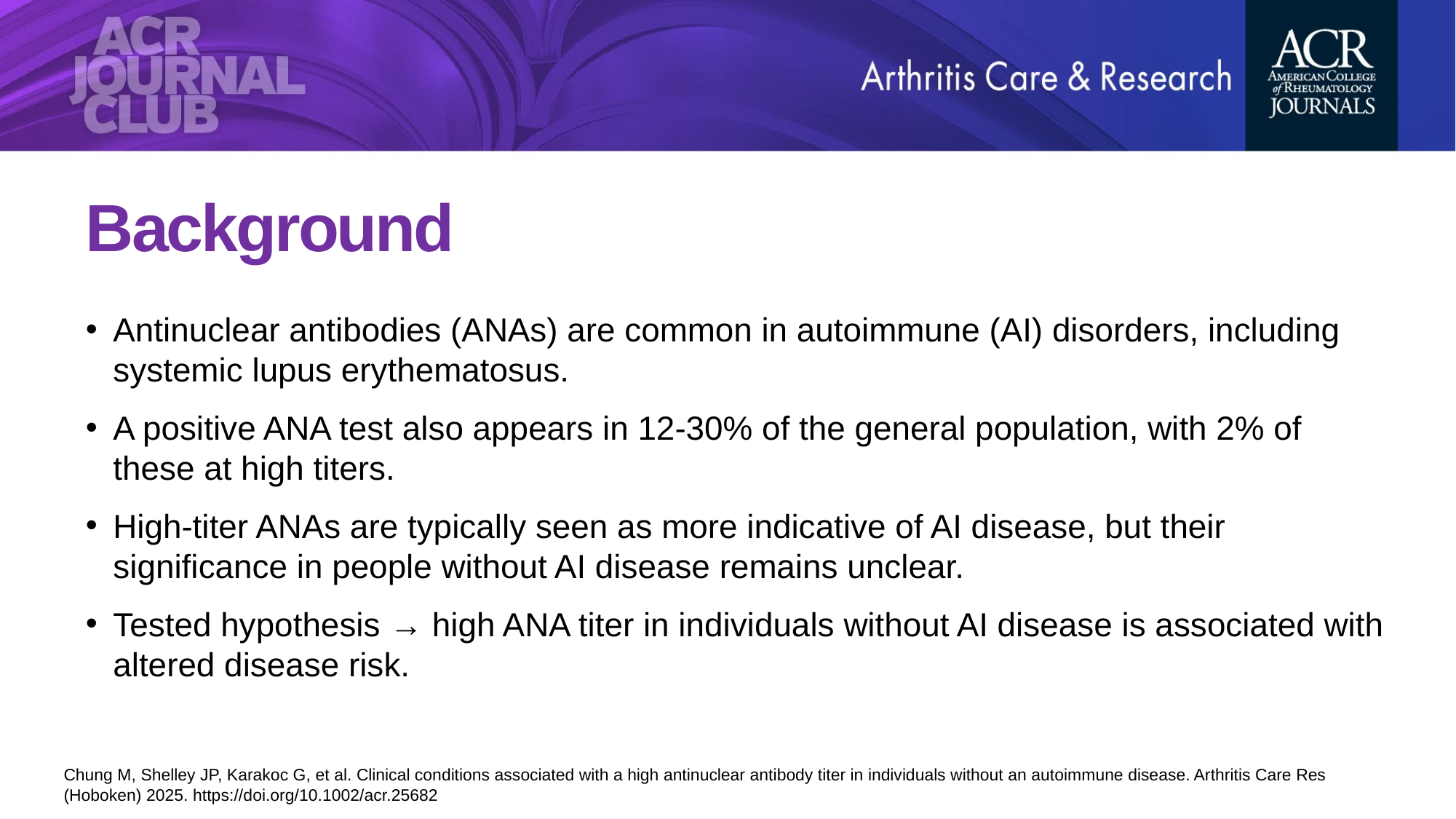

# Background
Antinuclear antibodies (ANAs) are common in autoimmune (AI) disorders, including systemic lupus erythematosus.
A positive ANA test also appears in 12-30% of the general population, with 2% of these at high titers.
High-titer ANAs are typically seen as more indicative of AI disease, but their significance in people without AI disease remains unclear.
Tested hypothesis → high ANA titer in individuals without AI disease is associated with altered disease risk.
Chung M, Shelley JP, Karakoc G, et al. Clinical conditions associated with a high antinuclear antibody titer in individuals without an autoimmune disease. Arthritis Care Res (Hoboken) 2025. https://doi.org/10.1002/acr.25682

## Slide 3
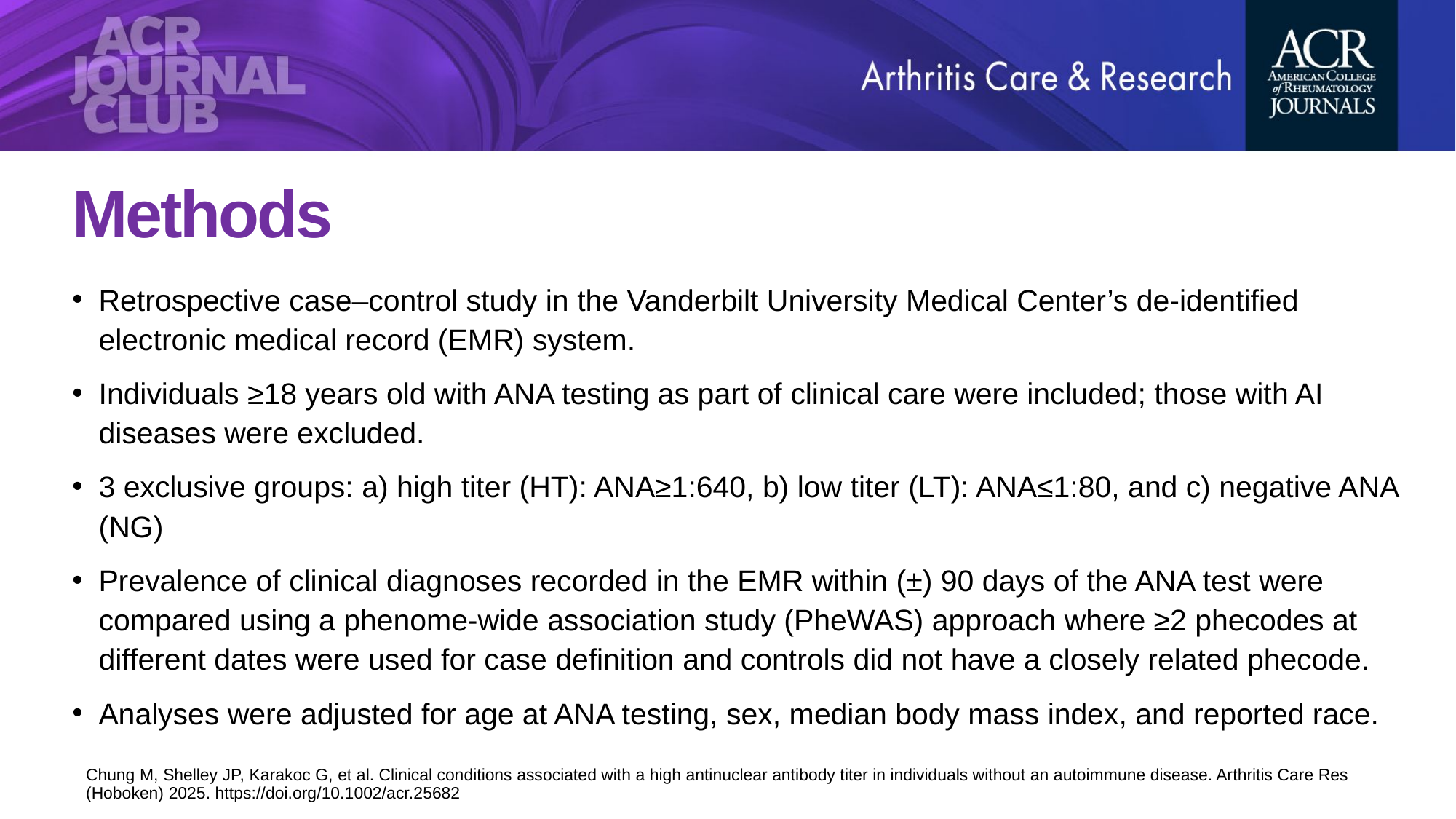

# Methods
Retrospective case–control study in the Vanderbilt University Medical Center’s de-identified electronic medical record (EMR) system.
Individuals ≥18 years old with ANA testing as part of clinical care were included; those with AI diseases were excluded.
3 exclusive groups: a) high titer (HT): ANA≥1:640, b) low titer (LT): ANA≤1:80, and c) negative ANA (NG)
Prevalence of clinical diagnoses recorded in the EMR within (±) 90 days of the ANA test were compared using a phenome-wide association study (PheWAS) approach where ≥2 phecodes at different dates were used for case definition and controls did not have a closely related phecode.
Analyses were adjusted for age at ANA testing, sex, median body mass index, and reported race.
Chung M, Shelley JP, Karakoc G, et al. Clinical conditions associated with a high antinuclear antibody titer in individuals without an autoimmune disease. Arthritis Care Res (Hoboken) 2025. https://doi.org/10.1002/acr.25682

## Slide 4
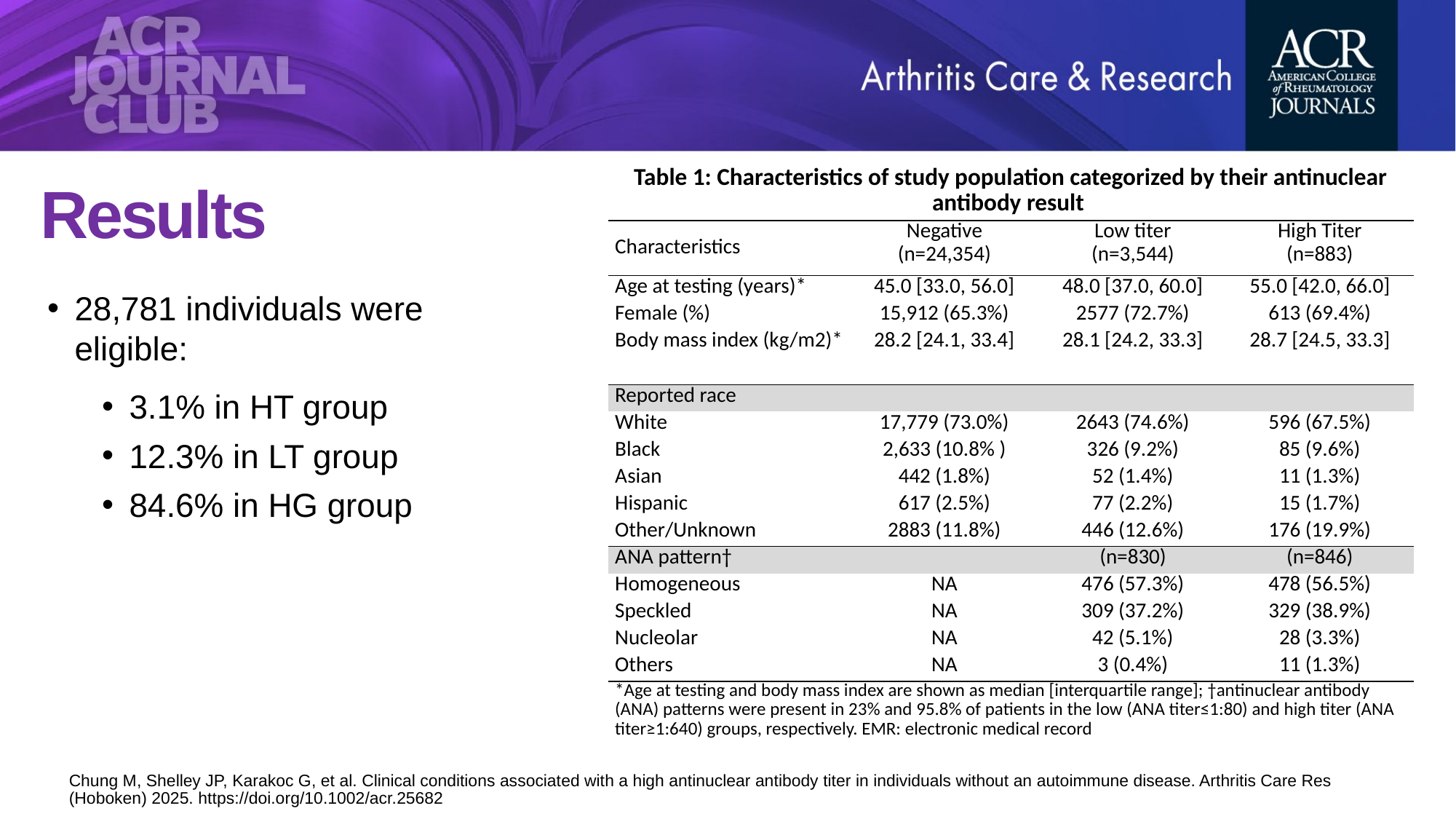

# Results
| Table 1: Characteristics of study population categorized by their antinuclear antibody result | | | |
| --- | --- | --- | --- |
| Characteristics | Negative (n=24,354) | Low titer (n=3,544) | High Titer (n=883) |
| Age at testing (years)\* | 45.0 [33.0, 56.0] | 48.0 [37.0, 60.0] | 55.0 [42.0, 66.0] |
| Female (%) | 15,912 (65.3%) | 2577 (72.7%) | 613 (69.4%) |
| Body mass index (kg/m2)\* | 28.2 [24.1, 33.4] | 28.1 [24.2, 33.3] | 28.7 [24.5, 33.3] |
| Reported race | | | |
| White | 17,779 (73.0%) | 2643 (74.6%) | 596 (67.5%) |
| Black | 2,633 (10.8% ) | 326 (9.2%) | 85 (9.6%) |
| Asian | 442 (1.8%) | 52 (1.4%) | 11 (1.3%) |
| Hispanic | 617 (2.5%) | 77 (2.2%) | 15 (1.7%) |
| Other/Unknown | 2883 (11.8%) | 446 (12.6%) | 176 (19.9%) |
| ANA pattern† | | (n=830) | (n=846) |
| Homogeneous | NA | 476 (57.3%) | 478 (56.5%) |
| Speckled | NA | 309 (37.2%) | 329 (38.9%) |
| Nucleolar | NA | 42 (5.1%) | 28 (3.3%) |
| Others | NA | 3 (0.4%) | 11 (1.3%) |
| \*Age at testing and body mass index are shown as median [interquartile range]; †antinuclear antibody (ANA) patterns were present in 23% and 95.8% of patients in the low (ANA titer≤1:80) and high titer (ANA titer≥1:640) groups, respectively. EMR: electronic medical record | | | |
28,781 individuals were eligible:
3.1% in HT group
12.3% in LT group
84.6% in HG group
Chung M, Shelley JP, Karakoc G, et al. Clinical conditions associated with a high antinuclear antibody titer in individuals without an autoimmune disease. Arthritis Care Res (Hoboken) 2025. https://doi.org/10.1002/acr.25682

## Slide 5
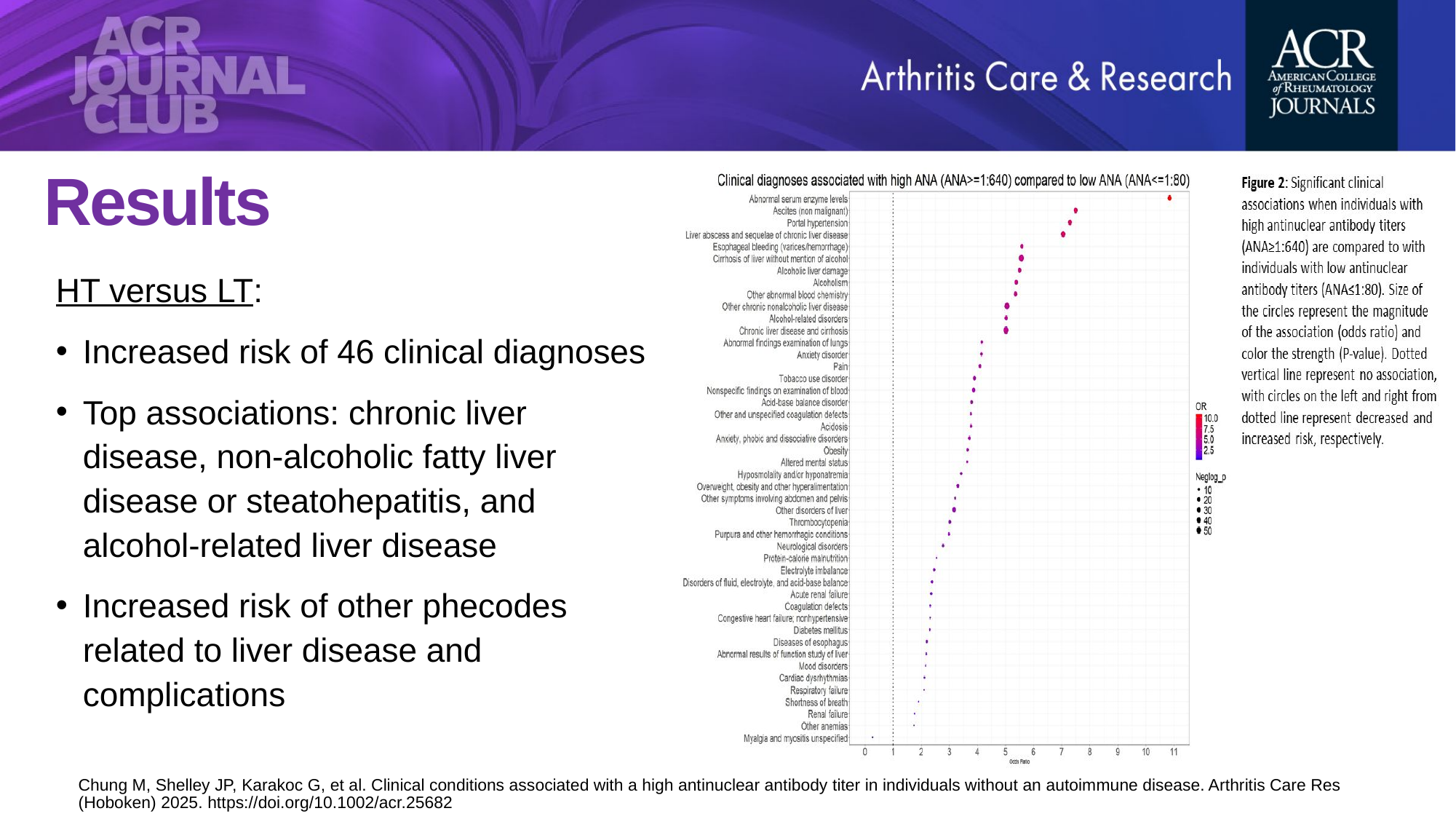

# Results
HT versus LT:
Increased risk of 46 clinical diagnoses
Top associations: chronic liver disease, non-alcoholic fatty liver disease or steatohepatitis, and alcohol-related liver disease
Increased risk of other phecodes related to liver disease and complications
Chung M, Shelley JP, Karakoc G, et al. Clinical conditions associated with a high antinuclear antibody titer in individuals without an autoimmune disease. Arthritis Care Res (Hoboken) 2025. https://doi.org/10.1002/acr.25682

## Slide 6
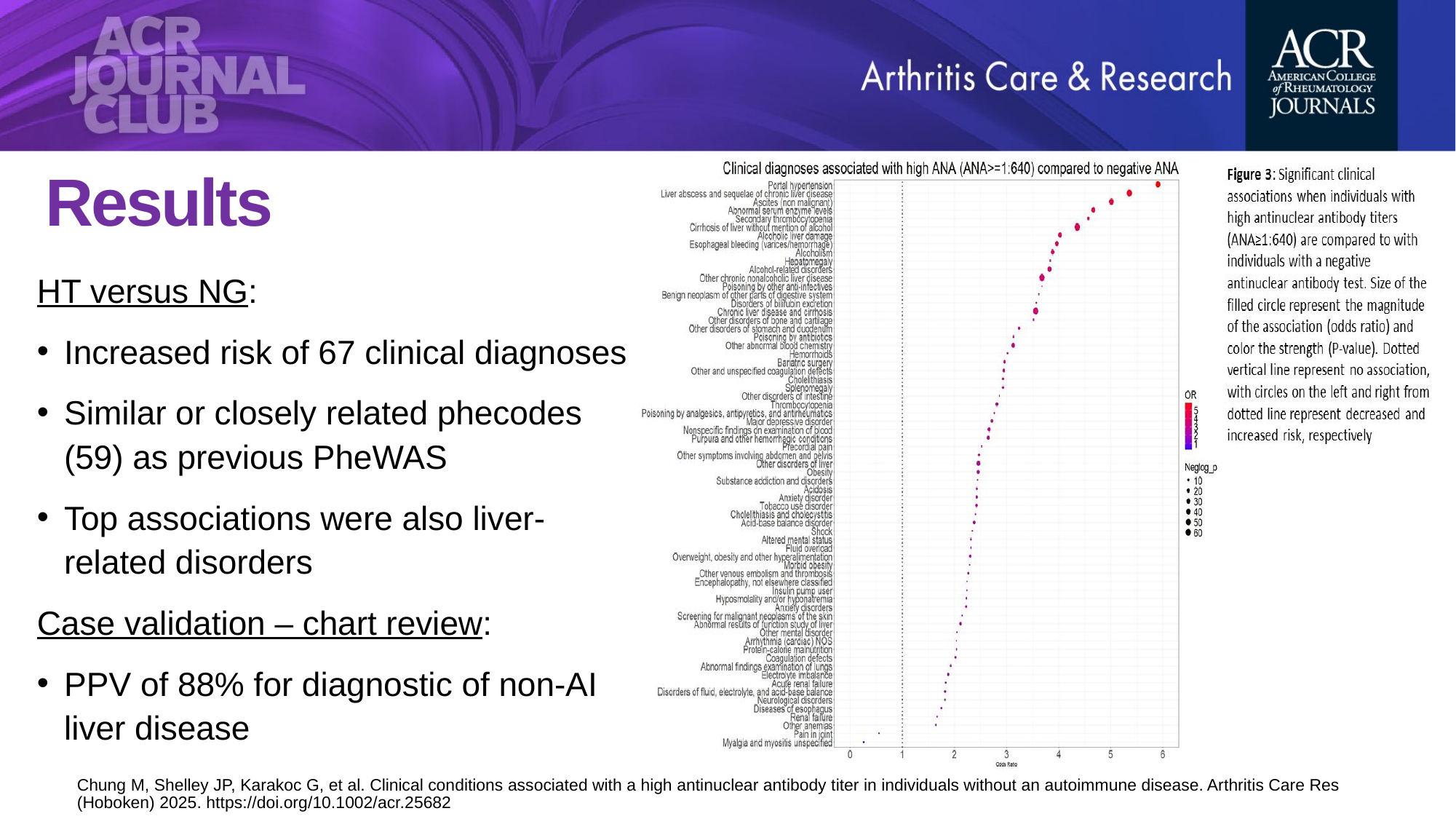

# Results
HT versus NG:
Increased risk of 67 clinical diagnoses
Similar or closely related phecodes (59) as previous PheWAS
Top associations were also liver- related disorders
Case validation – chart review:
PPV of 88% for diagnostic of non-AI liver disease
Chung M, Shelley JP, Karakoc G, et al. Clinical conditions associated with a high antinuclear antibody titer in individuals without an autoimmune disease. Arthritis Care Res (Hoboken) 2025. https://doi.org/10.1002/acr.25682

## Slide 7
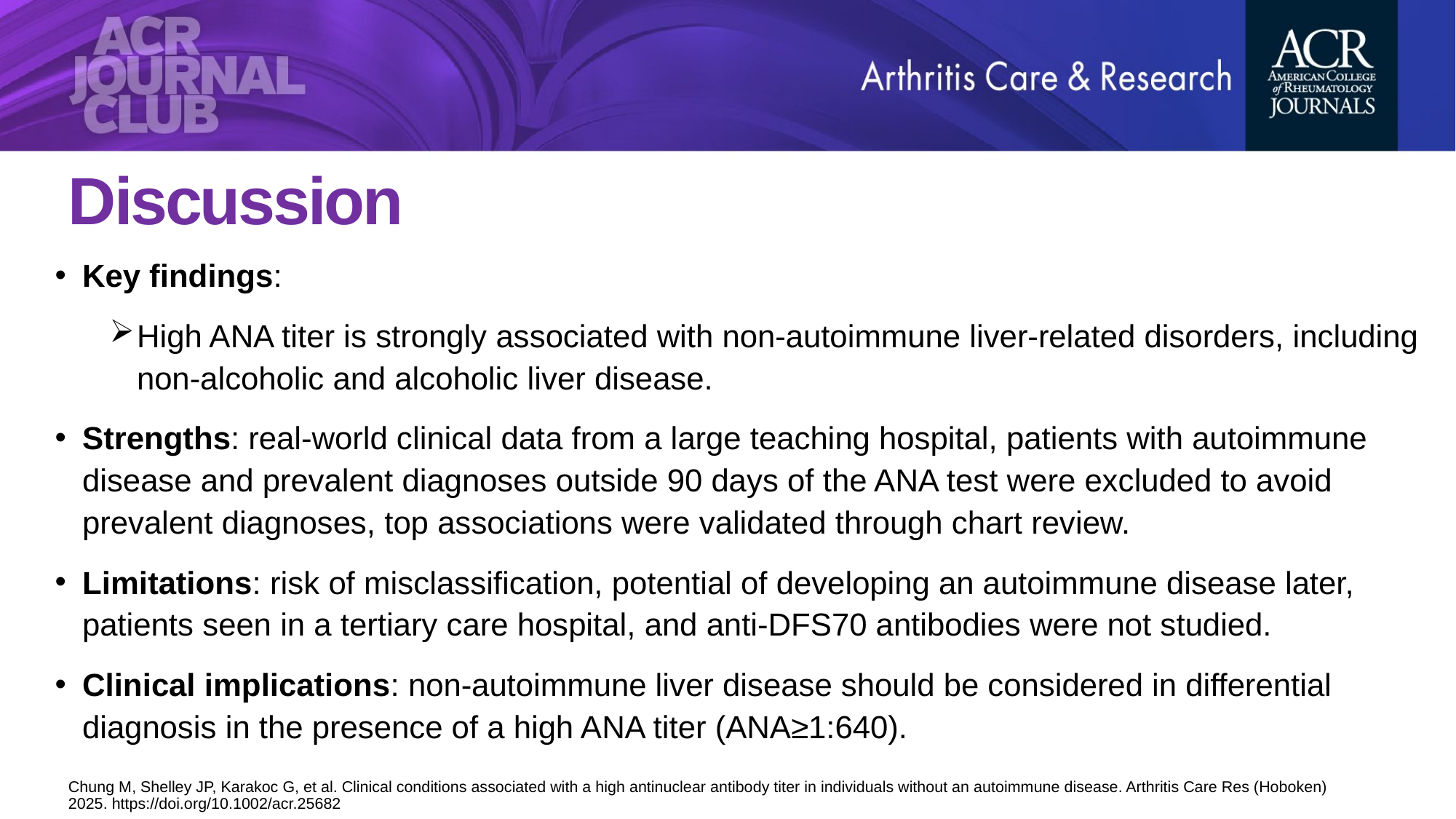

# Discussion
Key findings:
High ANA titer is strongly associated with non-autoimmune liver-related disorders, including non-alcoholic and alcoholic liver disease.
Strengths: real-world clinical data from a large teaching hospital, patients with autoimmune disease and prevalent diagnoses outside 90 days of the ANA test were excluded to avoid prevalent diagnoses, top associations were validated through chart review.
Limitations: risk of misclassification, potential of developing an autoimmune disease later, patients seen in a tertiary care hospital, and anti-DFS70 antibodies were not studied.
Clinical implications: non-autoimmune liver disease should be considered in differential diagnosis in the presence of a high ANA titer (ANA≥1:640).
Chung M, Shelley JP, Karakoc G, et al. Clinical conditions associated with a high antinuclear antibody titer in individuals without an autoimmune disease. Arthritis Care Res (Hoboken) 2025. https://doi.org/10.1002/acr.25682

## Slide 8
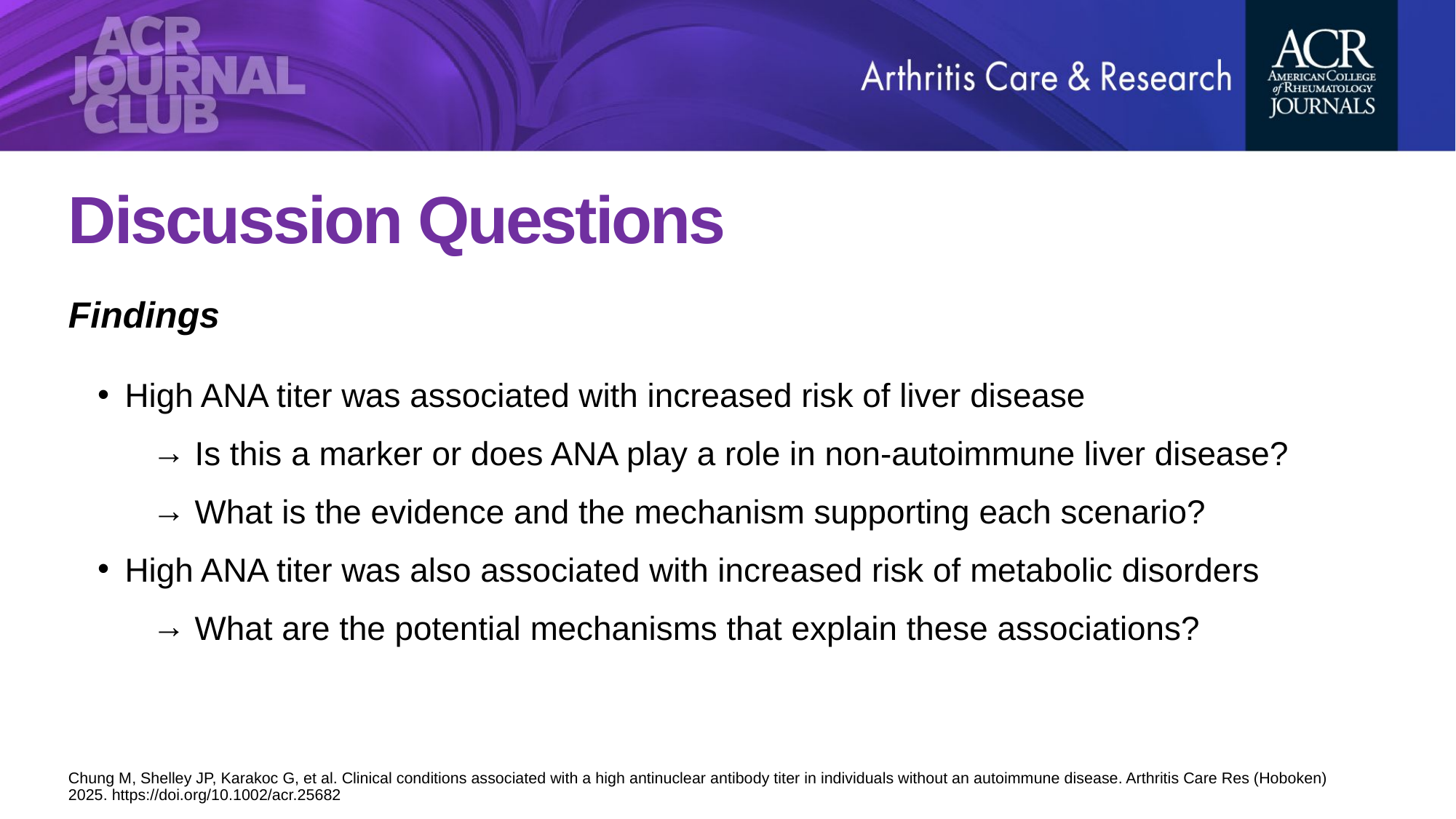

# Discussion Questions
Findings
High ANA titer was associated with increased risk of liver disease
 Is this a marker or does ANA play a role in non-autoimmune liver disease?
 What is the evidence and the mechanism supporting each scenario?
High ANA titer was also associated with increased risk of metabolic disorders
 What are the potential mechanisms that explain these associations?
Chung M, Shelley JP, Karakoc G, et al. Clinical conditions associated with a high antinuclear antibody titer in individuals without an autoimmune disease. Arthritis Care Res (Hoboken) 2025. https://doi.org/10.1002/acr.25682

## Slide 9
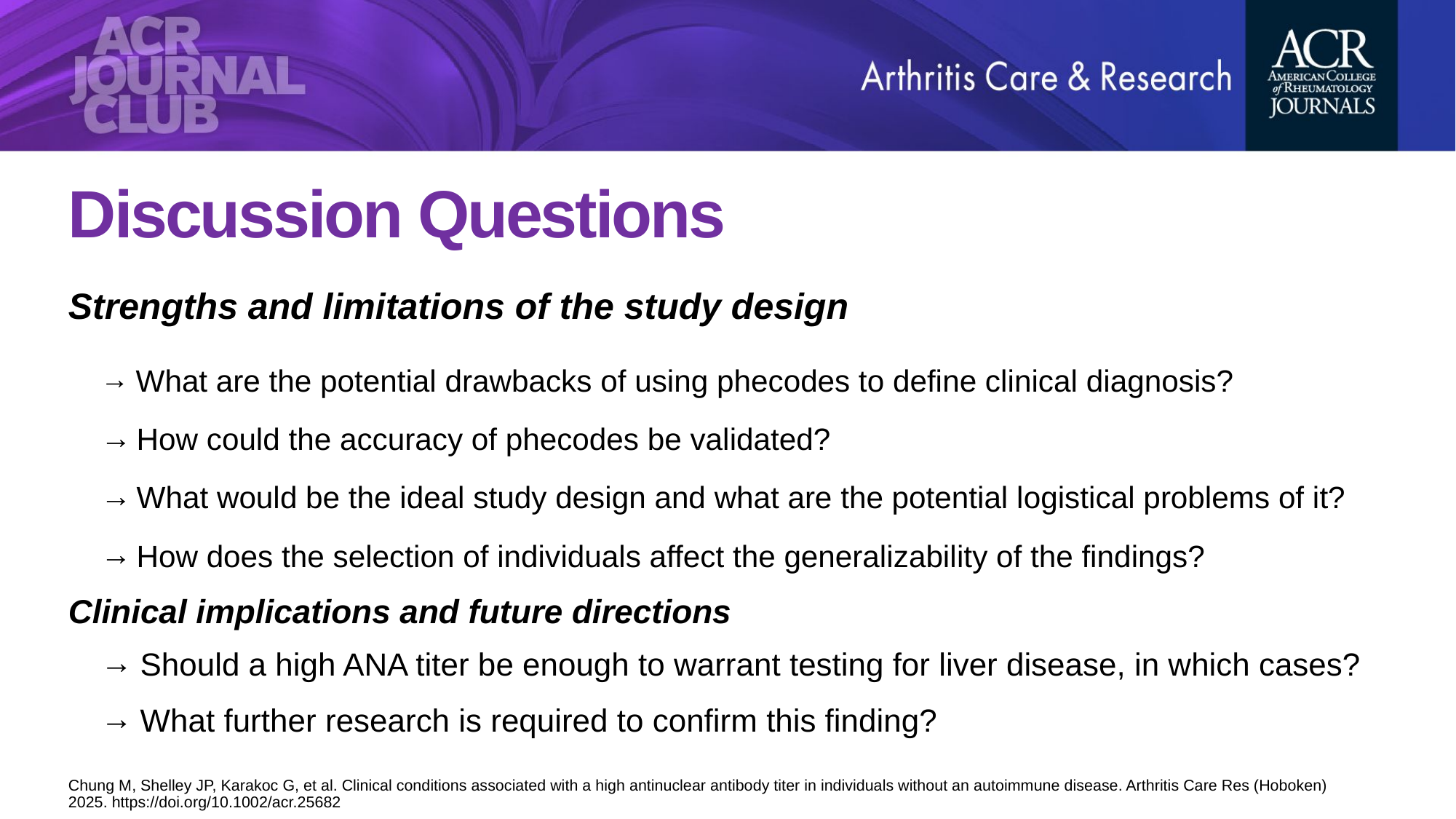

# Discussion Questions
Strengths and limitations of the study design
 What are the potential drawbacks of using phecodes to define clinical diagnosis?
 How could the accuracy of phecodes be validated?
 What would be the ideal study design and what are the potential logistical problems of it?
 How does the selection of individuals affect the generalizability of the findings?
Clinical implications and future directions
 Should a high ANA titer be enough to warrant testing for liver disease, in which cases?
 What further research is required to confirm this finding?
Chung M, Shelley JP, Karakoc G, et al. Clinical conditions associated with a high antinuclear antibody titer in individuals without an autoimmune disease. Arthritis Care Res (Hoboken) 2025. https://doi.org/10.1002/acr.25682
